# When is mesh fixation in TAPP-repair of primary inguinal hernia repair necessary? The register-based analysis of 11,230 cases

**DOI:** 10.1007/s00464-016-4754-8

**Published:** 2016-02-17

**Authors:** F. Mayer, H. Niebuhr, M. Lechner, A. Dinnewitzer, G. Köhler, M. Hukauf, R. H. Fortelny, R. Bittner, F. Köckerling

**Affiliations:** 1 Department of Surgery, Paracelsus Medical University, Müllner Hauptstrasse 48, 5020 Salzburg, Austria; 2 Hanse Hernia Center, Hamburg, Germany; 3 Department of General and Visceral Surgery, Sisters of Charity Hospital, Linz, Austria; 4 StatConsult GmbH, Magdeburg, Germany; 5 Department of General, Visceral and Oncological Surgery, Wilhelminenspital, Vienna, Austria; 6 Hernia Center, Winghofer Medicum, Rottenburg am Neckar, Germany; 7 Department of Surgery and Center of Minimally Invasive Surgery, Academic Teaching Hospital of Charité Medical School, Vivantes Hospital Spandau, Neue Bergstrasse 6, 13585 Berlin, Germany

**Keywords:** Inguinal hernia, TAPP, Mesh fixation, Medial hernia, Recurrence

## Abstract

Whereas for TEP the guidelines do not recommend mesh fixation on the basis of meta-analyses regardless of the defect size, for TAPP mesh fixation can be omitted only up to a defect size of 3 cm because of the paucity of studies on this topic. Hence, this study now seeks to explore this subject on the basis of prospective data from the Herniamed Hernia Registry. In the period September 01, 2009, to January 31, 2014, 11,228 male patients were operated on with the TAPP technique for a primary unilateral inguinal hernia and were followed up for 1 year. Mesh fixation was used for 7422 (66.1 %) of these patients and no mesh fixation for 3806 patients (33.9 %). Unadjusted analysis did not find any significant difference in the recurrence rate (0.88 % with fixation vs. 1.1 % without fixation; *p* = 0.259). Multivariable analysis of all potential influence factors (age, ASA, BMI, risk factors, defect size, mesh fixation, localization of defect, mesh size) did not identify any factor that impacted recurrence on 1-year follow-up. Only for medial and combined defect localization versus lateral localization was a highly significant effect identified (*p* < 0.001). With mesh fixation and larger mesh size, it was possible to significantly reduce the recurrence rate for larger medial hernias in this series (*p* = 0.046). For TAPP repair of an inguinal hernia, mesh fixation is not necessary in a significant number of patients. Patients with a medial and combined hernia are at higher risk of recurrence. In the patient series analyzed, it was possible to significantly reduce the recurrence rate with mesh fixation and larger mesh size for medial defects.

The longstanding standard practice for TAPP was to use mesh fixation with tackers to prevent recurrence [1]. But atraumatic mesh fixation fibrin sealants are being increasingly employed to prevent chronic pain in the wake of traumatic fixation methods [2]. Numerous studies have attested to the excellent results in terms of the recurrence rate achieved with fibrin sealants for atraumatic mesh fixation [3–6]. Comparative studies then explored, in particular for the total extraperitoneal patchplasty (TEP), whether mesh fixation could be completely dispensed with [7, 8]. In the guidelines for laparoscopic (TAPP) and endoscopic (TEP) treatment of inguinal hernia of the International Endohernia Society (IEHS), a statement with level of evidence 1 B pointed out that fixation and non-fixation of the mesh were associated with equally low recurrence rates in both TAPP and TEP [9]. However, in most studies the hernia opening was small (<3 cm) or not measured [9]. Therefore, the guidelines recommended that when using TAPP or TEP techniques non-fixation could be considered for types L I, II, and M I, II hernias (EHS classification) [9]. For TAPP and TEP repair of big defects (L III, M III), the mesh should be fixed [9]. In an update of the Guidelines of the International Endohernia Society, ten new studies with evidence level 1 have been included. For TEP, with evidence level 1 A, these stated that fixation and non-fixation of the mesh in TEP were associated with an equal risk of recurrence [10]. For TAPP, the recommendations remained unchanged. Hence, in the case of TAPP it remained unclear whether mesh fixation was needed to prevent recurrence, at least for defect sizes >3 cm (EHS classification L III, M III). Therefore, this paper now seeks to explore this subject on the basis of prospective data of the Herniamed Hernia Registry.

## Patients and methods

As of March, 19, 2015, 426 participating hospitals and office-based surgeons mainly from Germany, Austria, and Switzerland had entered prospective data into the multicenter internet-based Herniamed Hernia Registry on their patients who had undergone hernia surgery [11]. This present study analyzed the prospective data collected for all male patients who had been operated on with an endoscopic TAPP technique for repair of a primary unilateral inguinal hernia in the period September 01, 2009, up to and including January 31, 2014. On 1-year follow-up, the general practitioner and patients were asked by questionnaire about any recurrences. Only those patients for whom 1-year follow-up results were available were included in the analysis. Other inclusion criteria included: age >16 years and medial/lateral/combined types of inguinal hernia based on the EHS classification [12]. In total, 11,228 patients were included in uni- and multivariate analysis for investigation of the impact of mesh fixation as well as of other potential influence factors impacting onset of a recurrence during the 1-year follow-up of TAPP operation. Details of all enrolled patients regarding the documented hernia defect size are given in Table 1 and of the fixation method in Table 2. During the observation period, 7422 patients (66.1 %) were operated on while using mesh fixation and 3806 patients (33.9 %) without mesh fixation.

**Table 1:** Distribution of defect size and fixation/non-fixation

	Size of defect						Total	
	I (<1.5 cm)		II (1.5–3 cm)		III (>3 cm)			
	*n*	%	*n*	%	*n*	%	*n*	%
Mesh fixation	852	56.76	4652	62.91	1918	82.25	7422	66.10
No mesh fixation	649	43.24	2743	37.09	414	17.75	3806	33.90
Total	1501	100.00	7395	100.00	2332	100.00	11,228	100.00

**Table 2:** Distribution of defect size in the group with mesh fixation and fixation type

	Size of defect						Total	
	I (<1.5 cm)		II (1.5–3 cm)		III (>3 cm)			
	*n*	%	*n*	%	*n*	%	*n*	%
Type of fixation								
Suture	121	14.20	760	16.34	446	23.25	1327	17.88
Tacker	393	46.13	2219	47.70	956	49.84	3568	48.07
Glue	331	38.85	1607	34.54	468	24.40	2406	32.42
Combination	7	0.82	66	1.42	48	2.50	121	1.63
Total	852	100.00	4652	100.00	1918	100.00	7422	100.00

All analyses were performed with the software SAS 9.2 (SAS institute Inc., Cary, NY, USA) and intentionally calculated to a full significance level of 5 %, i.e., they were not corrected in respect of multiple tests, and each *p* value ≤ 0.05 represents a significant result. Unadjusted analyses were carried out to analyze how any individual influence variable affected an outcome parameter. For categorical target (outcome) variables, Fisher’s exact test was applied. For continuous target variables that followed the normal distribution, the robust *t* test (Satterthwaite) was used.

To eliminate the effect of any confounders arising from different characteristics related to the patient or surgical technique, the results of unadjusted analysis were verified once again in multivariable analysis. In addition to fixation (yes/no), it was also possible to simultaneously review all the other influence factors.

The binary regression model for dichotomous target variables was used to identify the influence of the various factors in multivariable analysis. Odds ratios (OR) and corresponding 95 % confidence intervals based on the Wald test are given for estimates. For influence variables with more than two categories, one of these values was used in each case as a reference category. For the continuous variable age (years), the 10-year odds ratio is given, for BMI (kg/m^2^) a five-point odds ratio, and for mesh size the ten-point odds ratio. The results are sorted on the basis of influence and presented in tabular form.

## Results

### Unadjusted results

Unadjusted analysis of the groups compared, i.e., TAPP with versus without mesh fixation, revealed, in some cases, significant differences in the patient characteristics and hernia findings (Table 3). The patients in the mesh fixation group were significantly older (57.4 years ± 14.8 vs. 54.4 years ± 15.7 [mean ± STD], *p* < 0.001), and larger meshes were used (151.1 cm^2^ ± 19.3 vs. 145.8 cm^2^ ± 15.6 [mean ± STD], *p* < 0.001). For large hernia defects (EHS III), the mesh was fixed significantly more often (82.2 % with mesh fixation vs. 17.8 % without mesh fixation) (Table 1). Likewise, for a medial hernia the implanted mesh was fixed significantly more often (30.8 % with mesh fixation vs. 24.9 % without mesh fixation; *p* < 0.001) (Table 3). A clear difference was identified between the two groups with regard to the presence of at least one risk factor (*p* = 0.011). A large proportion, at 25.8 %, of patients without mesh fixation had at least one relevant risk factor compared with those without mesh fixation, at 23.6 % (*p* = 0.001). That was also true for nicotine abuse (12.4 % without mesh fixation vs. 8.7 % with mesh fixation; *p* < 0.001).

**Table 3:** Demographic and surgery-related data

		Mesh fixation	No mesh fixation	*p*
Age (years)	Mean ± STD	57.4 ± 14.8	54.4 ± 15.7	<0.001
BMI (kg/m²)	Mean ± STD	25.9 ± 3.4	25.9 ± 3.3	0.573
Mesh size (cm²)	Mean ± STD	151.1 ± 19.3	145.8 ± 15.6	<0.001

	*n* (7422)	%	*n* (3806)	%	*p*
ASA					
I	2601	35.04	1282	33.68	0.027
II	3994	53.81	2037	53.52	
III/IV	827	11.14	487	12.80	
Defect size (EHS)					
I (<1.5 cm)	852	11.48	649	17.05	<0.001
II (1.5–3 cm)	4652	62.68	2743	72.07	
III (>3 cm)	1918	25.84	414	10.88	
Localization of defect (EHS)					
Medial (M)	2285	30.79	948	24.91	<0.001
Lateral (L)	4477	60.32	2298	60.38	
Combined (C)	660	8.89	560	14.71	
*Risk factors*					
Overall					
Yes	1749	23.57	980	25.75	0.011
No	5673	76.43	2826	74.25	
COPD					
Yes	321	4.32	196	5.15	0.051
No	7101	95.68	3610	94.85	
Diabetes					
Yes	318	4.28	164	4.31	0.961
No	7104	95.72	3642	95.69	
Aortic aneurysm					
Yes	22	0.30	9	0.24	0.705
No	7400	99.70	3797	99.76	
Immunosuppression					
Yes	34	0.46	14	0.37	0.544
No	7388	99.54	3792	99.63	
Corticoids					
Yes	50	0.67	23	0.60	0.711
No	7372	99.33	3783	99.40	
Smoking					
Yes	643	8.66	470	12.35	<.001
No	6779	91.34	3336	87.65	
Coagulopathy					
Yes	74	1.00	37	0.97	1.000
No	7348	99.00	3769	99.03	
Antiplatelet medication					
Yes	525	7.07	206	5.41	<0.001
No	6897	92.93	3600	94.59	
Cumarin medication					
Yes	133	1.79	52	1.37	0.100
No	7289	98.21	3754	98.63	

Demographic parameters (Table 3) are demonstrated in relation to fixation/non-fixation and include the age of the patients (years), BMI (kg/m^2^), size of the mesh implant (cm^2^), ASA score (I–IV), size of the hernia defect (EHS I–III), localization of the hernia defect (medial-M/lateral-L/combined-C; EHS classification), and hernia-specific risk factors

Unadjusted analysis of the relationship between mesh fixation and non-fixation for TAPP did not reveal any significant difference in the recurrence rate on 1-year follow-up (Table 4). The recurrence rate was 0.9 % in the mesh fixation group and 1.1 % in the non-fixation group (*p* = 0.259).

**Table 4:** Unadjusted analysis of the recurrence rates on 1-year follow-up

	Mesh fixation		No mesh fixation		*p*
	*n*	%	*n*	%	
Recurrent hernia (1-year follow-up: 100 %)					
Yes	65	0.88	42	1.10	0.259
No	7357	99.12	3764	98.90	

### Multivariable analysis

In this multivariable analysis (Table 5), all potential influence factors were reviewed with regard to onset of a recurrence. No relevant influence was identified for mesh fixation compared with non-fixation (*p* = 0.399). That was also true for the defect size (*p* = 0.383), with no significant difference observed on comparing defect sizes >3 cm (EHS classification III) with sizes <1.5 cm (EHS classification I) and 1.5–3 cm (EHS classification II). Nor did the mesh size have any significant impact on onset of recurrence. For the patient-related influence factors such as age, ASA score, BMI value, the risk factors COPD, and smoking as well as the other risk factors, multivariable analysis did not identify any effect on onset of recurrence. The only factor that had a highly significant impact on recurrence was hernia localization (*p* < 0.001). Whereas a lateral hernia was associated with a lower probability of onset of recurrence, a medial inguinal hernia and a combined hernia with a medial portion presented a highly significantly higher risk for onset of recurrence (*p* < 0.001). With a prevalence of 0.9 % for the entire patient collective, this would correspond to five recurrences for every 1000 operations of hernias with lateral EHS localization compared with 11 recurrences for patients with medial EHS localization. Hence, medial and combined hernias constitute a highly significant risk factor for onset of recurrence following TAPP, but that was not true for patient-related factors, hernia size, and mesh non-fixation.

**Table 5:** Multivariable analysis of recurrence (model fit: *p* = 0.004)

Parameter	*p* value	Variables	OR	95 %-CI	
Localization of defect (EHS)	<0.001	Combined versus medial	1.137	0.656	1.970
		Lateral versus medial	0.463	0.303	0.707
Risk factors: COPD/smoking	0.097	Yes versus no	0.556	0.278	1.111
BMI (five-point OR)	0.109		1.240	0.953	1.613
Size of mesh (ten-point OR)	0.192		0.929	0.832	1.038
Size of defect (EHS)	0.383	I (<1.5 cm) versus III (>3 cm)	1.330	0.694	2.546
		II (1.5–3 cm) versus III (>3 cm)	0.914	0.558	1.499
Fixation of mesh	0.399	No fixation versus fixation	1.194	0.791	1.800
Risk factors (others)^a^	0.408	Yes versus no	1.269	0.721	2.234
ASA	0.720	II versus I	1.106	0.683	1.791
		III/IV versus I	1.352	0.650	2.812
Age [10-year OR]	0.869		1.013	0.868	1.183

^a^Risk factors (others): immunosuppression, antiplatelet medication, coagulopathy, diabetes, corticoids, anticoagulation, aortic aneurysm

### Subgroup analysis

If, in view of the results of multivariable analysis, one compares the recurrence rates in unadjusted analysis in relation to the EHS localization, highly significant differences unfavorable to medial and combined hernias are seen (Table 6). If one then checks the role of fixation in the medial inguinal hernia group, which is at higher risk of recurrence, one notes that it was possible to significantly reduce the recurrence rate with mesh fixation (Table 7). No significant difference was found in the recurrence rate between the various fixation techniques (tacker, glue, suture, combination) (Table 8). In addition, where mesh fixation was used to repair medial inguinal hernias, a significantly larger mesh size was used (Table 9). Besides, analysis of the meshes used for at least 5 % of medial inguinal hernias demonstrated significant differences (Table 10). For example, one notable finding was that in the group with no mesh fixation a greater number of self-adhesive, titanized, and 3D standard meshes were used (Table 10). Since the medial sac reduction is not documented in the Herniamed Registry, no conclusions on its implications can be drawn from the data presented here.

**Table 6:** Comparision of recurrence rates depending on EHS localization

	Medial		Lateral		Combined		*p*
	*n*	%	*n*	%	*n*	%	
Recurrence							
Yes	44	1.36	44	0.65	19	1.56	<0.001
No	3190	98.64	6732	99.35	1201	1.56

**Table 7:** Comparision of recurrence rates in TAPP with and without mesh fixation in medial inguinal hernias

	Mesh fixation		No mesh fixation		*p*
	*n*	%	*n*	%	
Recurrence					
Yes	25	1.09	19	2.00	0.046
No	2260	98.91	929	98.00

**Table 8:** Comparision of recurrence rates in TAPP with mesh fixation depending on type of fixation in medial inguinal hernias

	Suture		Tacker		Glue		Combined		*p*
	*n*	%	*n*	%	*n*	%	*n*	%	
Recurrence									
Yes	4	0.97	16	1.34	5	0.79	0	0	0.746
No	407	99.03	1182	98.66	627	99.21	44	100.00

**Table 9:** Comparision of mesh sizes in TAPP with and without mesh fixation in medial inguinal hernias

		Fixation		*p*
		Mesh fixation (*n* = 2285)	No Mesh fixation (*n* = 948)	
Mesh size (cm^2^)	Mean ± STD	151.0 ± 21.1	145.2 ± 19.0	<0.001

**Table 10:** Comparision of meshes used in TAPP with and without mesh fixation in medial inguinal hernias

	Mesh fixation		No mesh fixation		*p*
	*n*	%	*n*	%	
Type of meshes					
Other <5 %	1079	47.22	416	43.88	<0.001
Ultrapro	604	26.43	80	8.44	
Parietene ProGrip	6	0.26	186	19.62	
TiMesh light	184	8.05	107	11.29	
3DMax Light Mesh	61	2.67	135	14.24	
Optilene Mesh LP	351	15.36	24	2.53	

## Discussion

This present analysis of data from the Herniamed Hernia Registry compared the recurrence rates on 1-year follow-up in respect of mesh fixation versus non-fixation in TAPP. Univariable analysis did not find any significant difference between these two parameters. However, since there were significant differences between the two groups in terms of their demographic and surgery-related data, multivariable analysis was performed to identify the influence factors that significantly impacted the recurrence rate on 1-year follow-up. The latter revealed that for TAPP, too, mesh fixation did not have any relevant impact on the recurrence rate regardless of the defect size. A similar conclusion was reported by a prospective randomized trial that compared 273 TAPP operations with mesh fixation versus 263 without mesh fixation [13].

Nor did multivariable analysis find any evidence that age, ASA score, BMI value, or patient-related risk factors exerted any influence on onset of recurrence. Here it must be pointed out that unlike one systematic review [14], no effect on recurrence rate was identified for patients with either COPD or nicotine abuse.

The only highly significant factor impacting onset of recurrence following TAPP for primary unilateral inguinal hernia repair in men was a medial or combined hernia based on the EHS classification. That finding was also confirmed in the systematic review by Burcharth et al. [14] which found that a direct inguinal hernia was found to be a risk factor for recurrence with a pooled RR of 1.91 (95 % CI 1.62–2.36; *p* < 0.001).

Unlike a lateral inguinal hernia, where the peritoneal hernia sac is removed from the inguinal canal and the inguinal canal closes curtain-like, additional surgical measures are necessary taken to repair the hernia defect for the medial inguinal hernia [9, 15–19]. The content of the direct hernia cavity, generally composed of preperitoneal fat, is dissected out, leaving the hernia cavity as a rigid outpouching of the transversalis fascia. Consequently, there is a higher risk of seroma for medial inguinal hernias following endoscopic repair [9, 15–19]. This medial hernia cavity is at also greater risk of recurrence since it represents more a bridging situation compared with the lateral inguinal hernia. Therefore, the requirements for adequate overlap are more stringent.

It is crucial when using an endoscopic technique (TEP, TAPP) to repair medial inguinal hernia that “complete medial sac reduction” be performed to avoid onset of seroma or recurrence. Since the lining of the medial hernia cavity is formed by the transversalis fascia outpouching, the latter is clasped and pulled inwards until the space is completely reduced (“complete medial sac reduction”) [9, 15–19]. Next, the transversalis fascia that has been pulled inwards is now either fixed with a suture to Cooper’s ligament or blocked off with a Roeder loop [18, 19]. The utmost attention should be paid to this technical step of “complete medial sac reduction” in both TAPP and TEP since it serves to prevent seromas as well as recurrence.

Moreover, in this situation it may be necessary to use a mesh size of 17 × 12 cm instead of the standard size of 15 × 10 cm. For example, analysis of the subgroup of medial inguinal hernias in the Herniamed Registry did indeed reveal that in the mesh fixation group significantly larger size meshes were used. By contrast, in the group with no mesh fixation a greater number of self-adhesive, titanized and 3D standard meshes were also used. The data presented here also demonstrate that for larger medial and combined hernias additional fixation of the mesh is needed using either properly placed absorbable tackers, sutures, or atraumatic fibrin sealants. The data also show that the type of fixation did not impact the recurrence rate.

In summary, it can be stated that for TAPP repair of an inguinal hernia fixation of the mesh is not needed in a significant number of patients. Patients with a medial and combined hernia are at a higher risk of recurrence. The choice of a greater mesh and “complete medial sac reduction” must be carefully made to obtain a plane inguinal region surface for mesh placement and greater mesh overlap. This helps to reduce both the recurrence and the seroma rates. The present study has demonstrated that on using mesh fixation for TAPP, regardless of whether with tacker, suture, glue, or combined, the recurrence rates for larger medial hernias were significantly lower.

## Appendix: Herniamed Study Group

### Scientific Board

**Köckerling**, Ferdinand (Chairman) (Berlin); **Berger**, Dieter (Baden-Baden); **Bittner**, Reinhard (Rottenburg); **Bruns**, Christiane (Magdeburg); **Dalicho**, Stephan (Magdeburg); **Fortelny**, René (Wien); **Jacob**, Dietmar (Berlin); **Koch**, Andreas (Cottbus); **Kraft,** Barbara (Stuttgart); **Kuthe**, Andreas (Hannover); **Lippert**, Hans (Magdeburg): **Lorenz**, Ralph (Berlin); **Mayer**, Franz (Salzburg); **Moesta**, Kurt Thomas (Hannover); **Niebuhr**, Henning (Hamburg); **Peiper**, Christian (Hamm); **Pross**, Matthias (Berlin); **Reinpold**, Wolfgang (Hamburg); **Simon**, Thomas (Sinsheim); **Stechemesser**, Bernd (Köln); **Unger**, Solveig (Chemnitz).

### Participants

**Ahmetov**, Azat (Saint-Petersburg); **Alapatt,** Terence Francis (Frankfurt/Main); **Anders,** Stefan (Berlin); **Anderson**, Jürina (Würzburg); **Arndt**, Anatoli (Elmshorn); **Asperger,** Walter (Halle); **Avram**, Iulian (Saarbrücken); **Barkus**; Jörg (Velbert); **Becker**, Matthias (Freital); **Behrend**, Matthias (Deggendorf); **Beuleke,** Andrea (Burgwedel); **Berger,** Dieter (Baden-Baden); **Bittner,** Reinhard (Rottenburg); **Blaha,** Pavel (Zwiesel); **Blumberg,** Claus (Lübeck); **Böckmann,** Ulrich (Papenburg); **Böhle**, Arnd Steffen (Bremen); **Böttger,** Thomas Carsten (Fürth); **Bolle**, Ludger (Berlin); **Borchert,** Erika (Grevenbroich); **Born**, Henry (Leipzig); **Brabender,** Jan (Köln); **Brauckmann,** Markus (Rüdesheim am Rhein); **Breitenbuch von**, Philipp (Radebeul); **Brüggemann**, Armin (Kassel); **Brütting**, Alfred (Erlangen); **Budzier**, Eckhard (Meldorf); **Burchett**, Bert (Waren); **Burghardt**, Jens (Rüdersdorf); **Carus**, Thomas (Bremen); **Cejnar**, Stephan-Alexander (München); **Chirikov**, Ruslan (Dorsten); **Comman,** Andreas (Bogen); **Crescent**i, Fabio (Verden/Aller); **Dapunt**, Emanuela (Bruneck); **Decker**, Georg (Berlin); **Demmel**, Michael (Arnsberg); **Descloux,** Alexandre (Baden); **Deusch**, Klaus-Peter (Wiesbaden); **Dick**, Marcus (Neumünster); **Dieterich**, Klaus (Ditzingen); **Dietz**, Harald (Landshut); **Dittmann**, Michael (Northeim); **Dornbusch**, Jan (Herzberg/Elster); **Drummer**, Bernhard (Forchheim); **Eckermann**, Oliver (Luckenwalde); **Eckhoff,** Jörn/Hamburg); **Elger**, Karlheinz (Germersheim); **Engelhardt**, Thomas (Erfurt); **Erichsen,** Axel (Friedrichshafen); **Eucker**, Dietmar (Bruderholz); **Fackeldey**, Volker (Kitzingen); **Farke**, Stefan (Delmenhorst); **Faust**, Hendrik (Emden); **Federmann**, Georg (Seehausen); **Feichter**, Albert (Wien); **Fiedler,** Michael (Eisenberg); **Fischer**, Ines (Wiener Neustadt); **Fortelny**, René H. (Wien); **Franczak**, Andreas (Wien); **Franke**, Claus (Düsseldorf); **Frankenberg von**, Moritz (Salem); **Frehner**, Wolfgang (Ottobeuren); **Friedhoff**, Klaus (Andernach); **Friedrich,** Jürgen (Essen); **Frings**, Wolfram (Bonn); **Fritsche**, Ralf (Darmstadt); **Frommhold,** Klaus (Coesfeld); **Frunder**, Albrecht (Tübingen); **Fuhrer**, Günther (Reutlingen); **Gassler,** Harald (Villach); **Gawad**, Karim A. Frankfurt/Main); **Gerdes**, Martin (Ostercappeln); **Germanov**, German (Halberstadt; **Gilg**, Kai-Uwe (Hartmannsdorf); **Glaubitz**, Martin (Neumünster); **Glutig,** Holger (Meissen); **Gmeiner**, Dietmar (Bad Dürrnberg); **Göring**, Herbert (München); **Grebe**, Werner (Rheda-Wiedenbrück); **Grothe**, Dirk (Melle); **Gürtler**, Thomas (Zürich); **Hache**, Helmer (Löbau); **Hämmerle**, Alexander (Bad Pyrmont); **Haffner**, Eugen (Hamm); **Hain**, Hans-Jürgen (Gross-Umstadt); **Hammans**, Sebastian (Lingen); **Hampe**, Carsten (Garbsen); **Harrer**, Petra (Starnberg); **Heinzmann**, Bernd (Magdeburg); **Heise**, Joachim Wilfried (Stolberg); **Heitland**, Tim (München); **Helbling**, Christian (Rapperswil); **Hempen**, Hans-Günther (Cloppenburg); **Henneking**, Klaus-Wilhelm (Bayreuth); **Hennes**, Norbert (Duisburg); **Hermes**, Wolfgang (Weyhe); **Herrgesell**, Holger (Berlin); **Herzing**, Holger Höchstadt); **Hessler**, Christian (Bingen); **Hildebrand**, Christiaan (Langenfeld); **Höferlin**, Andreas (Mainz); **Hoffmann**, Henry (Basel); **Hoffmann**, Michael (Kassel); **Hofmann**, Eva M. (Frankfurt/Main); **Hopfe**r, Frank (Eggenfelden); **Hornung**, Frederic (Wolfratshausen); **Hügel**, Omar (Hannover); **Hüttemann**, Martin (Oberhausen); **Huhn**, Ulla (Berlin); **Hunkeler**, Rolf (Zürich); **Imdahl**, Andreas (Heidenheim); **Jacob**, Dietmar (Berlin); **Jenert**, Burghard (Lichtenstein); **Jugenheimer**, Michael (Herrenberg); **Junger**, Marc (München); **Kaaden**, Stephan (Neustadt am Rübenberge); **Käs**, Stephan (Weiden); **Kahraman,** Orhan (Hamburg); **Kaiser,** Christian (Westerstede); **Kaiser**, Stefan (Kleinmachnow); **Kapischke**, Matthias (Hamburg); **Karch**, Matthias (Eichstätt); **Kasparek**, Michael S. (München); **Keck**, Heinrich (Wolfenbüttel); **Keller,** Hans W. (Bonn); **Kienzle**, Ulrich (Karlsruhe); **Kipfmüller**, Brigitte (Köthen); **Kirsch**, Ulrike (Oranienburg); **Klammer**, Frank (Ahlen); **Klatt**, Richard (Hagen); **Kleemann**, Nils (Perleberg); **Klein**, Karl-Hermann (Burbach); **Kleist**, Sven (Berlin); **Klobusicky**, Pavol (Bad Kissingen); **Kneifel**, Thomas (Datteln); **Knoop**, Michael (Frankfurt/Oder); **Knotter**, Bianca (Mannheim); **Koch,** Andreas (Cottbus); **Köckerling,** Ferdinand (Berlin); **Köhler**, Gernot (Linz); **König**, Oliver (Buchholz); **Kornblum**, Hans (Tübingen); **Krämer**, Dirk (Bad Zwischenahn); **Kraft**, Barbara (Stuttgart); **Kreissl**, Peter (Ebersberg); **Krones**, Carsten Johannes (Aachen); **Kruse,** Christinan (Aschaffenburg); **Kube**, Rainer (Cottbus); **Kühlberg**, Thomas (Berlin); **Kuhn,** Roger (Gifhorn); **Kusch,** Eduard (Gütersloh); **Kuthe,** Andreas (Hannover); **Ladberg**, Ralf (Bremen); **Ladra**, Jürgen (Düren); **Lahr-Eigen**, Rolf (Potsdam); **Lainka**, Martin (Wattenscheid); **Lammers**, Bernhard J. (Neuss); **Lancee**, Steffen (Alsfeld); **Lange**, Claas (Berlin); **Laps**, Rainer (Ehringshausen); **Larusson**, Hannes Jon (Pinneberg); **Lauschke**, Holger (Duisburg); **Leher,** Markus (Schärding); **Leidl**, Stefan (Waidhofen/Ybbs); **Lenz**, Stefan (Berlin); **Lesch**, Alexander (Kamp-Lintfort); **Liedke**, Marc Olaf (Heide); **Lienert**, Mark (Duisburg); **Limberger**, Andreas (Schrobenhausen); **Limmer**, Stefan (Würzburg); **Locher**, Martin (Kiel); **Loghmanieh**, Siawasch (Viersen); **Lorenz**, Ralph (Berlin); **Mallmann**, Bernhard (Krefeld); **Manger**, Regina (Schwabmünchen); **Maurer**, Stephan (Münster); **Mayer**, Franz (Salzburg); **Mellert**, Joachim (Höxter); **Menzel**, Ingo (Weimar); **Meurer**, Kirsten (Bochum); **Meyer**, Moritz (Ahaus**)**; **Mirow**, Lutz (Kirchberg); **Mittenzwey**, Hans-Joachim (Berlin); **Mörder-Köttgen**, Anja (Freiburg); **Moesta**, Kurt Thomas (Hannover); Moldenhauer, Ingolf (Braunschweig); **Morkramer**, Rolf (Xanten); **Mosa**, Tawfik (Merseburg); **Müller**, Hannes (Schlanders); **Münzberg**, Gregor (Berlin); **Mussack,** Thomas (St. Gallen); **Neumann**, Jürgen (Haan); **Neumeuer**, Kai (Paderborn); **Niebuhr,** Henning (Hamburg); **Nix**, Carsten (Walsrode); **Nölling**, Anke (Burbach); **Nostitz**, Friedrich Zoltán (Mühlhausen); **Obermaier**, Straubing); **Öz-Schmidt**, Meryem (Hanau); **Oldorf**, Peter (Usingen); **Olivieri**, Manuel (Pforzheim); **Pawelzik**, Marek (Hamburg); **Peiper**, Christian (Hamm); **Peitgen**, Klaus (Bottrop); **Pertl**, Alexander (Spittal/Drau); **Philipp**, Mark (Rostock); **Pickart**, Lutz (Bad Langensalza); **Pizzera**, Christian (Graz); **Pöllath**, Martin (Sulzbach-Rosenberg); **Possin**, Ulrich (Laatzen); **Prenzel**, Klaus (Bad Neuenahr-Ahrweiler); **Pröve**, Florian (Goslar); **Pronnet**, Thomas (Fürstenfeldbruck); **Pross**, Matthias (Berlin); **Puff**, Johannes (Dinkelsbühl); **Rabl**, Anton (Passau); **Rapp**, Martin (Neunkirchen); **Reck**, Thomas (Püttlingen); **Reinpold,** Wolfgang (Hamburg); **Reuter**, Christoph (Quakenbrück); **Richter,** Jörg (Winnenden); **Riemann**, Kerstin (Alzenau-Wasserlos); **Rodehorst**, Anette (Otterndorf); **Roehr**, Thomas (Rödental); **Roncossek**, Bremerhaven); **Roth** Hartmut (Nürnberg); **Sardoschau**, Nihad (Saarbrücken); **Sauer**, Gottfried (Rüsselsheim); **Sauer**, Jörg (Arnsberg); **Seekamp**, Axel (Freiburg); **Seelig**, Matthias (Bad Soden); **Seidel**, Hanka (Eschweiler); **Seiler**, Christoph Michael (Warendorf); **Seltmann,** Cornelia (Hachenburg); **Senkal,** Metin (Witten); **Shamiyeh**, Andreas (Linz); **Shang**, Edward (München); **Siemssen**, Björn (Berlin); **Sievers,** Dörte (Hamburg); **Silbernik**, Daniel (Bonn); **Simon**, Thomas (Sinsheim); **Sinn**, Daniel (Olpe); **Sinning**, Frank (Nürnberg); **Smaxwil**, Constatin Aurel (Stuttgart); **Schabel**, Volker (Kirchheim/Teck); **Schadd**, Peter (Euskirchen); **Schassen von**, Christian (Hamburg); **Schattenhofer**, Thomas (Vilshofen); **Scheidbach**, Hubert (Neustadt/Saale); **Schelp**, Lothar (Wuppertal); **Scherf**, Alexander (Pforzheim); **Scheyer**, Mathias (Bludenz); **Schimmelpenning,** Hendrik (Neustadt in Holstein); **Schinkel**, Svenja (Kempten); **Schmid**, Michael (Gera); **Schmid,** Thomas (Innsbruck); **Schmidt**, Rainer (Paderborn); **Schmidt**, Sven-Christian (Berlin); **Schmidt,** Ulf (Mechernich); **Schmitz**, Heiner (Jena); **Schmitz**, Ronald (Altenburg); **Schöche**, Jan (Borna); **Schoenen**, Detlef (Schwandorf); **Schrittwieser**, Rudolf/Bruck an der Mur); **Schroll**, Andreas (München); **Schultz**, Christian (Bremen-Lesum); **Schultz**, Harald (Landstuhl); **Schulze**, Frank P. Mülheim an der Ruhr); **Schumacher**, Franz-Josef (Oberhausen); **Schwab**, Robert (Koblenz); **Schwandner**, Thilo (Lich); **Schwarz**, Jochen Günter (Rottenburg); **Schymatzek**, Ulrich (Radevormwald); **Spangenberger**, Wolfgang (Bergisch-Gladbach); **Sperling**, Peter (Montabaur); **Staade**, Katja (Düsseldorf); **Staib**, Ludger (Esslingen); **Stamm**, Ingrid (Heppenheim); **Stark**, Wolfgang (Roth); **Stechemesser,** Bernd (Köln); **Steinhilper**, Uz (München); **Stengl**, Wolfgang (Nürnberg); **Stern**, Oliver (Hamburg); **Stöltzing**, Oliver (Meißen); **Stolte**, Thomas (Mannheim); **Stopinski,** Jürgen (Schwalmstadt); **Stubbe**, Hendrik (Güstrow/); **Stülzebach**, Carsten (Friedrichroda); **Tepel,** Jürgen (Osnabrück); **Terzić**, Alexander (Wildeshausen); **Teske,** Ulrich (Essen); **Thews**, Andreas (Schönebeck); **Tichomirow,** Alexej (Brühl); **Tillenburg**, Wolfgang (Marktheidenfeld); **Timmermann,** Wolfgang (Hagen); **Tomov**, Tsvetomir (Koblenz; **Train**, Stefan H. (Gronau); **Trauzettel**, Uwe (Plettenberg); **Triechelt**, Uwe (Langenhagen); **Ulcar**, Heimo (Schwarzach im Pongau); **Unger**, Solveig (Chemnitz); **Verweel**, Rainer (Hürth); **Vogel**, Ulrike (Berlin); **Voigt**, Rigo (Altenburg); **Voit**, Gerhard (Fürth); **Volkers**, Hans-Uwe (Norden); **Vossough**, Alexander (Neuss); **Wallasch**, Andreas (Menden); **Wallner**, Axel (Lüdinghausen); **Warscher,** Manfred (Lienz); **Warwas**, Markus (Bonn); **Weber**, Jörg (Köln); **Weihrauch**, Thomas (Ilmenau); **Weiß**, Johannes (Schwetzingen); **Weißenbach**, Peter (Neunkirchen); **Werner**, Uwe (Lübbecke-Rahden); **Wessel,** Ina (Duisburg); **Weyhe**, Dirk (Oldenburg); **Wieber**, Isabell (Köln); **Wiesmann**, Aloys (Rheine); **Wiesner**, Ingo (Halle); **Withöft**, Detlef (Neutraubling); **Woehe**, Fritz (Sanderhausen); **Wolf**, Claudio (Neuwied); **Yaksan**, Arif (Wermeskirchen); **Yildirim**, Selcuk (Berlin); **Zarras**, Konstantinos (Düsseldorf); **Zeller**, Johannes (Waldshut-Tiengen); **Zhorzel**, Sven (Agatharied); **Zuz**, Gerhard (Leipzig);
